# Alignment of vaccine codes using an ontology of vaccine descriptions

**DOI:** 10.1186/s13326-022-00278-0

**Published:** 2022-10-18

**Authors:** Benedikt FH Becker, Jan A Kors, Erik M van Mulligen, Miriam CJM Sturkenboom

**Affiliations:** Department of Medical Informatics, Erasmus University Medical Center, Dr. Molewaterplein 50, Rotterdam, 3015 GE The Netherlands

**Keywords:** Vaccines, Coding systems, Alignment, Ontology

## Abstract

**Background:**

Vaccine information in European electronic health record (EHR) databases is represented using various clinical and database-specific coding systems and drug vocabularies. The lack of harmonization constitutes a challenge in reusing EHR data in collaborative benefit-risk studies about vaccines.

**Methods:**

We designed an ontology of the properties that are commonly used in vaccine descriptions, called Ontology of Vaccine Descriptions (VaccO), with a dictionary for the analysis of multilingual vaccine descriptions. We implemented five algorithms for the alignment of vaccine coding systems, i.e., the identification of corresponding codes from different coding ystems, based on an analysis of the code descriptors. The algorithms were evaluated by comparing their results with manually created alignments in two reference sets including clinical and database-specific coding systems with multilingual code descriptors.

**Results:**

The best-performing algorithm represented code descriptors as logical statements about entities in the VaccO ontology and used an ontology reasoner to infer common properties and identify corresponding vaccine codes. The evaluation demonstrated excellent performance of the approach (F-scores 0.91 and 0.96).

**Conclusion:**

The VaccO ontology allows the identification, representation, and comparison of heterogeneous descriptions of vaccines. The automatic alignment of vaccine coding systems can accelerate the readiness of EHR databases in collaborative vaccine studies.

## Background

The ADVANCE project (Accelerated Development of VAccine beNe t-risk Collaboration in Europe) is building systems to provide best evidence to support decision-making on vaccination in Europe based on the reuse of electronic health record (EHR) data [1]. An important aspect is the extraction of vaccine exposure data from EHR databases across Europe, which use various coding systems to represent data. One challenge in reusing EHRs is the lack of harmonization between vaccine coding systems in EHR databases.

Vaccines are described in medical coding systems on different levels: as a product, as a pharmacologic group, or by characteristics in an ontology. The level of description determines which additional information is available about the recorded vaccine. First, a vaccine can be indicated on the level of individual products using its commercial or generic name or using a code from a normalized drug or vaccine terminology. Drug terminologies unify different names of vaccines and provide many product properties, e.g., ingredients and authorizations. Several such drug terminologies exist: The Article 57 database (Art57 DB) from the European Medicines Agency provides information about medical products authorized in Europe, including their composition, indications, and authorization details [2]. RxNorm from the US National Library of Medicine, and the National Drug Codes from the Food and Drug Administration (FDA) have a comparable scope of information for therapeutic drugs and vaccines authorized in the United States [3, 4].

Second, a vaccine can be recorded more generally by its pharmacologic group, which is common in coding systems. A code is defined by a short textual phrase – the code descriptor – that refers to the vaccine properties that are shared between group members, e.g., the disease or pathogen that a vaccine seeks to prevent (‘Influenza vaccines’ or ‘H1N1 vaccines’), or the vaccine strategy (‘attenuated vaccines’ or ‘inactivated vaccines’; we use the same property names as Plotkin where applicable [5]). Some coding systems possess a taxonomic hierarchy that subordinates codes representing more specific vaccine groups to codes representing more general groups. Only the information stated in the code descriptor and implied by the hierarchy is available about a recorded vaccine. Vaccine codes are defined in several medical coding systems including diagnosis coding systems (e.g., SNOMED Clinical Terms (SNOMED-CT) [6], Read-2 codes [7, 8], or Medical Subject Headings (MeSH) [9]), drug classification systems (e.g., Anatomical Therapeutic Chemical Classification System (ATC) [10]), and custom coding systems that may be specific to a particular EHR database and often using non-English code descriptors [11]. Some coding systems comprise codes in a taxonomic hierarchy and codes for individual vaccines (e.g., the National Drug File Reference Terminology (NDF-RT) [12, 13] and British National Formulary (BNF) [14]).

Third, vaccines can be represented by statements in an ontology. An ontology is an unambiguous definition of the entities and relations in a domain (‘the explicit specification of a conceptualization’) [15, 16]. Individuals and collections in a domain are represented by classes that are defined by common properties of the belonging individuals. The classes in the domain of vaccines may represent vaccines (individual products and vaccine groups), immunization targets, ingredients, manufacturers, and market authorizations. Properties of a vaccine can be inferred from the information available in the ontology. The Vaccine Investigation and Online Netwok (VIOLIN) maintains the Vaccine Ontology (VO), to date the most comprehensive ontology of immunological information about vaccines, with the objectives of standardizing data and enabling computer-assisted reasoning about vaccines in the United States and Canada [17]. VIOLIN provides several tools to access information about vaccines, including vaccine components, mechanisms, vaccine design, and literature [18, 19].

Currently, vaccine benefit-risk studies that utilize vaccine information from EHR databases with different coding systems have to go through a tedious manual semantic harmonization process to align the codes [20]. An automatic alignment of vaccine coding systems would accelerate the readiness to obtain information from the EHR databases for vaccine benefit-risk studies.

Various approaches have been proposed for aligning ontologies in general [21], medical coding systems [22–27], and drug coding systems [28, 29]. These approaches commonly use lexical, instance-based, or hierarchical information about codes and classes. However, not all approaches are applicable to the alignment of the vaccine coding systems used in EHR databases. Lexical techniques create alignments based on lexical comparison of code descriptors, which is unsuitable for coding systems with descriptors in different languages. For instance-based techniques, the similarity of two classes is asserted by comparing the instances that belong to each class, but coding systems usually do not contain information about the membership of individual products to vaccine codes. Hierarchical techniques employ the taxonomic hierarchy of the ontology, but vaccine coding systems used in EHR databases are often not hierarchically structured.

Codes in general drug coding systems are commonly defined by chemical structure, therapeutic intent, physiologic effect, mechanism of action, and pharmacokinetics [30, 31]. The predominant property category for defining vaccine classes is the immunization target (corresponding to the therapeutic intent), but vaccine strategies (corresponding to the production method) and administration routes, which are used in the definitions of vaccine codes, are uncommon in general drug coding systems. These differences between descriptors in general drug coding systems and descriptors in vaccine coding systems further hamper the transfer of algorithms for aligning drug coding systems to vaccine coding systems.

In this article, we describe and evaluate an automatic approach to the alignment of vaccine coding systems based on their (potentially multilingual) code descriptors. For this purpose we developed the Ontology of Vaccine Descriptions (VaccO) that models properties used in descriptors of vaccine codes, which contrast to the immunological properties of vaccines modelled in existing ontologies. Our alignment approach analyses code descriptors and represents vaccine properties in the VaccO ontology, and applies an ontology reasoner to identify codes with corresponding descriptors.

## Methods

### Construction of the VaccO ontology

A vaccine code in a medical coding system stands for an individual vaccine product or for a pharmacologic group of vaccines. To prepare the creation of the VaccO ontology, we first identified categories of the properties used to define the vaccine groups in a number of general, drug-specific, and custom, database-specific coding systems: SNOMED-CT, Read-2, MeSH, ATC, BNF, and Additional Health Data (AHD) from the database of the The Health Improvement Network (THIN).

Immunization targets (i.e., vaccine-preventable diseases and their pathogens) were used in all coding systems for the definition of vaccine codes (Table 1). Vaccine-preventable diseases and pathogens may be used interchangeably to describe equivalent vaccine groups (e.g., ‘Vaccine against cervical cancer’ and ‘Human papillomavirus vaccine’). Vaccine codes were further defined based on vaccine strategies, ingredients (including adjuvants, excipients, and active ingredients), routes of administration, and valences (which can denote the number of pathogen strains targeted by a vaccine or the number of components in combination vaccines).

**Table 1:** Categories of properties used in vaccine descriptions. A check mark ($\checkmark$) indicates that a property category (row) is used for defining vaccine codes in a coding system (column)

Prop. category	SNOMED-CT	Read-2	MeSH	ATC	BNF	AHD
Pathogen	$\checkmark$	$\checkmark$	$\checkmark$	$\checkmark$	$\checkmark$	$\checkmark$
Disease	$\checkmark$	$\checkmark$	$\checkmark$	$\checkmark$	$\checkmark$	$\checkmark$
Strategy	$\checkmark$	$\checkmark$	$\checkmark$	$\checkmark$		$\checkmark$
Ingredient		$\checkmark$	$\checkmark$		$\checkmark$	$\checkmark$
Route		$\checkmark$	$\checkmark$	$\checkmark$		$\checkmark$
Valence		$\checkmark$	$\checkmark$	$\checkmark$		

The VaccO ontology is specified using the Web Ontology Language (OWL2) [32]. Classes are hierarchically structured by the subclass relation (*is-a*) and their extension is specified by expressions of description logic (DL) describing the properties of the class [33]. For example, the class of influenza vaccines can be defined by the DL expression *Vaccine* that *immunizes-against* *Influenza*, where *Vaccine* and *Influenza* refer to other classes and *immunizes-against* is a property^1^. A class can further contain one or more terms to state the meaning of the class in free text.

**Fig. 1:**
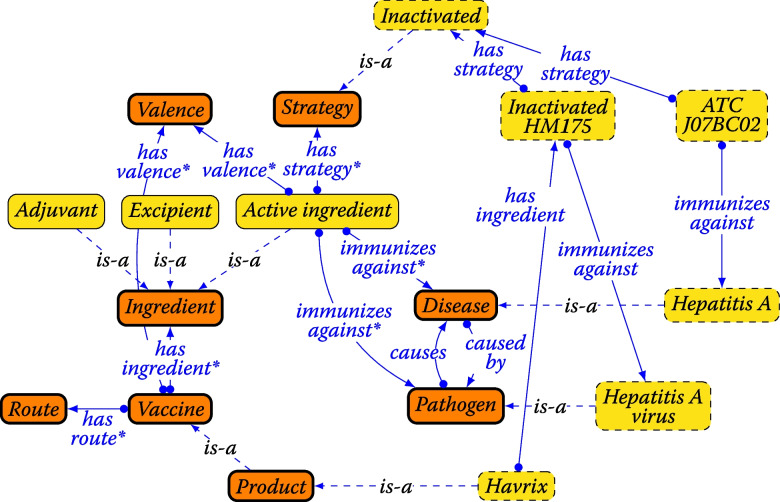
Structure of the core VaccO ontology. Fundamental classes representing property categories are shown as orange boxes. Properties marked with an asterisk are propagated along subclass relations (*is-a*) and containment relations (*has-ingredient*). Their domains are expanded along the same relations. Examples for representing a vaccine product from the Article 57 database (‘Havrix’), and a vaccine group defined by ATC code J07BC02 (‘Hepatitis A, inactivated’) are shown with dashed frames. The visualization follows the Graffoo specification [45]

The categories of vaccine properties, vaccines, and vaccine products are represented by fundamental classes, which lay out the overall structure of the VaccO ontology: *Vaccine, Valence, Route, Ingredient, Strategy, Disease*, and *Pathogen* (see Fig. 1). Classes for pharmacological groups and vaccine products are defined as subclasses of *Vaccine*. The other classes in the VaccO ontology and their English terms were compiled from the following resources (by manual analysis if not stated differently):

- Classes for vaccine products and their ingredients were extracted from the Art57 DB using a Python script.
- Common pharmacological vaccine groups and their abbreviations (such as ‘DTaP’) were identified in vaccine literature [5, 34–37] and a monograph from the US Centers for Disease Control and Prevention [38].
- Vaccine strategies and terms were extracted from descriptions in literature, classes in the VO ontology, and vaccine codes in MeSH.
- Indications of drugs including immunization targets of vaccines are not defined in any publicly available, formalized resource to the best of our knowledge. We extracted classes for pathogens and diseases, and causal relationships between them instead from the descriptions of MeSH headings (‘scope notes’). Terms were automatically compiled from the codes that the Unified Medical Language System [39] links to the MeSH headings of pathogens and diseases in the following coding systems: Consumer Health Vocabulary (CHV) [40], International Statistical Classification of Diseases, 10th Revision, Clinical Revision [41], Medical Dictionary for Regulatory Activities [42], MeSH, the taxonomy of the National Center for Biotechnology Information [43], and SNOMED-CT.
- Administration routes were identified in the Art57 DB and the VO ontology, and terms (including common abbreviations) were compiled from literature and a monograph of the FDA [44].
- Classes and terms for valences (‘1-valent’ up to ‘30-valent’) were generated automatically, and common terms for valence 1-10 were added manually (e.g., ‘pentavalent’).

Relations between classes are expressed in OWL2 using (existential) object properties. An object property is defined by its domain and by its range. For example, the domain of the object property *has-ingredient* is the class *Vaccine* and its range is the class *Ingredient*. Other object properties in VaccO are *immunizes-against* (relating *Vaccine* and *Active ingredient* with *Pathogen* and *Disease*), *has-strategy* (relating *Vaccine* and *Active-ingredient* with *Strategy*), *has-valence* (relating *Vaccine* with *Valence*), and *has-route* (relating *Vaccine* with *Route*), *causes* (relating *Pathogen* with *Disease*), and *caused-by* (relating *Disease* with *Pathogen*). Property chains were defined to allow for propagating properties from ingredients to containing vaccines, and to unify pathogens and diseases as immunization targets when they are in a causal relation (Table 2). For example, the property chain *has-ingredient* $\circ$ *immunizes-against* $\Rightarrow$ *immunizes-against* states that if a vaccine has an ingredient that immunizes against a specific target (left-hand side), the vaccine immunizes also against the target (right-hand side).

**Table 2:** Example inferences about compiled vaccine classes using property chains in VaccO: the propagation of the a) immunization targets and b) vaccine strategies from the active ingredients to vaccines, and c) the definition of immunization targets interchangeably by pathogen and vaccine-preventable diseases

	Available information	Property chain	Inferred information
a)	*v is-a Vaccine* that *has-ingredient I*. *I is-a Active-ingredient* that *imm.-against Flu*.	*has-ingred.* $\circ$ *imm.-against* $\Rightarrow$ *imm.-against*.	*v is-a Vaccine* that *imm.-against Flu*.
b)	*v is-a Vaccine* that *has-ingredient I*. *I is-a Active-ingredient* that *has-strategy Inactivated*.	*has-ingred.* $\circ$ *has-strategy* $\Rightarrow$ *has-strategy*.	*v is-a Vaccine* that *has-strategy Inactivated*.
c)	*v is-a Vaccine* that *imm.-against Hib*. *Hib is-a Pathogen* that *causes Cervical-cancer*.	*imm.-against* $\circ$ *causes* $\Rightarrow$ *imm.-against*.	*v* *is-a Vaccine* that *imm.-against Cervical cancer*.

### Representation of vaccine descriptions in VaccO

The representation of vaccine descriptions in VaccO involves three steps: The identification of vaccine properties in the free-text description, the compilation of the vaccine properties into logical expressions in the ontology, and the normalization of the comprised information as property values.

#### Identification of vaccine properties in free text

The set of all terms assigned to the classes in an ontology is called the ontology dictionary. The VaccO ontology dictionary constitutes the basis for identifying references to its classes in free text. Each occurrence of a term from the dictionary in an input text is considered a reference to the associated class. We refer to the set of classes identified in an input text *t* as *C*(*t*). For example, the input text $t=$ ‘Live/attenuated inuenza vaccine’ contains references to the classes in $C(t)=\{{Influenza}, {Attenuated}\}$.

We prepared the dictionary of VaccO for multilingual input by automatically translating all English terms using GoogleTranslate to Spanish, Italian, and Catalan (the languages of the vaccine code descriptors in the ADVANCE data sources) [46]. The multilingual dictionary is stored in the Apache Solr text search platform, and a Solr plugin for dictionary-based concept identification, Solr TextTagger, is used to identify occurrences of terms from the ontology dictionary in free text [47, 48].

#### Compilation of vaccine properties into the VaccO class

The representation of vaccine descriptions in VaccO is based on the compilation of a VaccO class *c* identified in the descriptor to a DL expression describing a vaccine, $[\![ c ]\!]$. The compilation depends on the category of *c* and corresponds to *c* itself if it is a vaccine (a class being a DL expression), or to the class of vaccines with a specific property if *c* is a vaccine property:

$$\begin{aligned}{}[\![ c ]\!] :=\left\{ \begin{array}{ll} c &{} \mathrm {if}\ c\ is-a\ Vaccine \\ Vaccine\ \mathrm {\underline{that}}\ has-strategy\ c &{} \mathrm {if}\ c\ is-a\ Strategy \\ Vaccine \ \mathrm {\underline{that}}\ immunizes-against\ c &{} \mathrm {if}\ c\ is-a\ Pathogen\ or\ Disease \\ Vaccine \ \mathrm {\underline{that}}\ has-ingredient\ c &{} \mathrm {if}\ c\ is-a\ Ingredient \\ Vaccine \ \mathrm {\underline{that}}\ has-valence\ c &{} \mathrm {if}\ c\ is-a\ Valence \\ Vaccine\ \mathrm {\underline{that}}\ has-route\ c &{} \mathrm {if}\ c\ is-a\ Route \end{array}\right. \end{aligned}$$

For example, the disease class *Tuberculosis* is compiled to the DL expression *Vaccine* that *immunizes-against* *Tuberculosis*. A set of classes is compiled into the conjunction of the compiled individual classes, $[\![\left\{ c_1, \ldots , c_n\right\} ]\!] := [\![ c_1 ]\!] \ \mathrm {\underline{and}}\ \ldots \ [\![ c_n ]\!]$.

A textual description *t* of a vaccine is represented by the compiled vaccine class $V(t) := [\![ C(t)]\!]$, defined by the result of compiling the classes identified in the description. For example, the vaccine class for the descriptor ‘Live/attenuated influenza vaccine’ is defined by the DL expression *Vaccine* that *immunizes-against Influenza* and *has-strategy* *Attenuated*.

#### Normalization to property values

The property values *P*(*t*) of a vaccine description *t* are an assignment of each object property in VaccO (*immunizes-against, has-route*, etc.) to all subclasses of the property range that conform to the vaccine description and the information available in VaccO. Formally, the property values *P*(*t*) contain for each property *p* all subclasses *c* in the range of *p*, where $\mathrm {VaccO} \vDash [\![ C(t) ]\!] \sqsubseteq \mathrm {{Vaccine}}\ \mathrm {\underline{that}}\ p\ c$ (using the notation by Baader [33]). For example, the property values for the descriptor ‘DTwP’ are [*immunizes-against*: {*Diphtheria, Tetanus, Pertussis*}; *has-strategy*: {*Inactivated*}].

The compiled vaccine class links information from the vaccine description with information in the VaccO ontology. An ontology reasoner is required to access information implied by the ontology, and the comparison of two compiled vaccine classes can only assess specification, generalization, or equivalence. However, the property values are an explicit representation of all information about a vaccine description implied by the ontology, and they can be compared with each other more flexibly using similarity measures for sets. Furthermore, equivalent vaccine descriptions based on pathogens (‘Influenza virus vaccine’), diseases (‘Flu vaccine’), abbreviations (‘IIV3’), or products (‘Influvac’) are normalized to the same property value [*immunizes-against*: {*Influenza*}].

The representation of vaccine classes and the conversion to property values was implemented in Java using the the OWL2 application programming interface and the JFact ontology reasoner [49, 50].

**Fig. 2:**
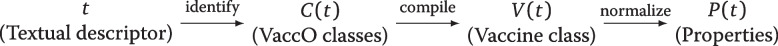
Pipeline for representing a textual vaccine description *t* using the VaccO ontology

Figure 2 summarizes the pipeline for representing a textual vaccine description using the VaccO ontology.

### Automatic code alignment and evaluation

An alignment between a source coding system and a target coding system assigns each source code to its closest corresponding target code. Our algorithm for creating an alignment first scores the similarity between each source code and each target code (where 1 indicates maximal similarity and 0 indicates no similarity). The target code with the highest similarity score is then assigned to the source code, provided that the score was larger than a preset similarity threshold. If the maximum score does not reach the threshold, no target code is assigned. If multiple target codes have the same maximum similarity score larger than the threshold, all target codes are assigned unless the target coding system has a taxonomic hierarchy. In that case, only the most general target codes with maximum similarity are assigned.

#### Alignment methods

**Fig. 3:**
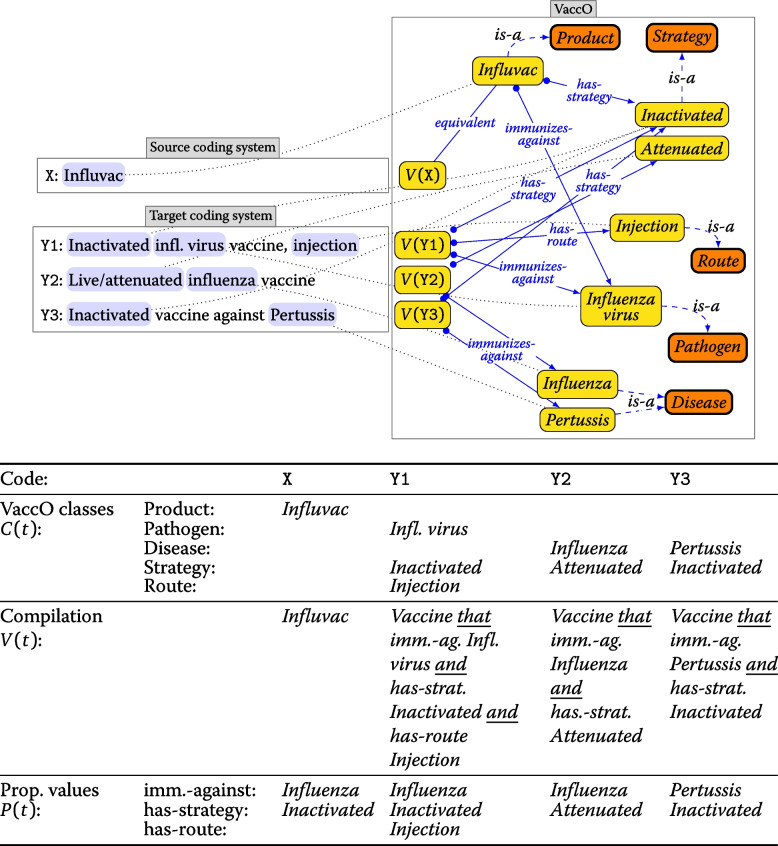
Example compilation of the textual descriptors *t* of vaccine codes X, Y1, Y2, and Y3 into classes in VaccO. Above: VaccO classes are identified in the code descriptors (blue boxes in the source and target code descriptors on the left) and compiled into vaccine classes (*V*(*X*), *V*(*Y*1), ...). Below: Representation of the vaccine descriptors in the VaccO similarity methods. The classes identified in the descriptor of code X do not overlap with those in the descriptors of codes Y1, Y2, or Y3, and the DL-expressions are not equivalent, resulting in a similarity of 0 for similarity methods Classes and Equivalence and a missing alignment for X. However, property values of code X and the target codes overlap, and X is assigned in Properties to code Y1, which has maximal similarity with X (Y1: 0.5, Y2: 0.3, Y3: 0)

We evaluated our alignment algorithm using two baseline similarity methods and three similarity methods involving the representation of vaccine descriptions in VaccO as described above. Example alignments for the VaccO -based methods are shown in Fig. 3.

- Method Tokens implemented a simple lexical technique. Each code descriptor was tokenized, and the similarity between two codes was measured by the Jaccard coefficient of the two sets of tokens. The Jaccard coefficient of two sets $s$ and $t$ is defined as $\left| s \cap t \right| /\left| s \cup t \right|$.
- Method Metamap used the MetaMap program to identify UMLS concept unique identiers (CUIs) for each code descriptor, abstracting over word inflections and synonyms [51]. MetaMap used a dictionary of English terms, and thus can only find concepts in English text. Similarity was defined by the Jaccard coefficient of the two sets of CUIs.
- Method Classes represented a code with descriptor $t$ as the set of classes identified in the code descriptor, $C(t)$. Similarity was defined by the Jaccard coefficient of the classes of the source code and the classes of the target code.
- Method Equivalence represented a code with descriptor $t$ by the compiled vaccine class, $V(t)$. Similarity between two codes was 1 if their compiled vaccine classes are equivalent and 0 otherwise. Assessing equivalence involved information implied from the VaccO ontology and is checked using the ontology reasoner.
- Method Properties represented a code with descriptor $t$ by its property values, $P(t)$. The similarity between a source code and target code was defined as 0 if the values of property *immunizes-against* differed, and by the overlap between the property values otherwise. The overlap was defined as the Jaccard coefficient between the property values.

#### Reference mappings

To evaluate our code alignment algorithm, we used two reference sets with manually curated alignments (Table 3). The first reference set Vactype used the Vactype coding system as a target. Vactype was developed as a pragmatic solution to harmonize the vaccine descriptors in the databases that participated in an early vaccine studies of the ADVANCE project [20]. It used English descriptors, and currently comprises 43 codes (for 28 single immunization targets with strategies, and 15 combinations). The Vactype reference set used five custom vaccine coding systems with multilingual descriptors from European EHR databases as source coding systems: the Catalonian Information System for Research in Primary Care (SIDIAP) with Catalan descriptors [52], the Spanish Base de datos para la Investigación Farmacoepidemiológica en Atencióon Primaria (BIFAP) with Spanish descriptors [53], the Italian paediatric database Pedianet with both English and Italian descriptors [54], and the regional primary care database of Venetia with Italian descriptors. The alignments in the Vactype reference set were manually created and validated by the database custodians in a proof-of-concept study of the ADVANCE project [20].

**Table 3:** Vaccine coding systems, languages, and number of source codes in the reference sets

Target	Source	Language	Codes
Vactype	Vactype	English	43
	BIFAP	Spanish	761
	SIDIAP	Catalan	98
	Venetia	English	21
	Pedianet-en	English	9
	Pedianet-it	Italian	9
Atc	ATC	English	114
	NDF-RT	English	40
	CHV	English	26
	MeSH	English	23
	VANDF	English	18
	CVX	English	18

The second reference set Atc comprised alignments from coding systems in the UMLS to the ATC target coding system. As of 2017, the ATC system contained 114 vaccine codes (with prefix J07). The coding systems with the largest number of mappings to ATC vaccine codes in the UMLS were used as source coding systems in the ATC reference set: Veterans A air National Drug File (VANDF), MeSH, CHV, Vaccine Administered (CVX), and NDF-RT. We corrected 17 code assignments where the source codes were not assigned to the most specific, corresponding ATC code in the UMLS.

Reflexive alignments in which either Vactype or ATC was both the source coding system and the target coding system were included in the evaluation to assess the completeness of the intermediate representation used by the different similarity methods.

#### Performance measures

The comparison of an automatically generated alignment with a reference alignment is based on the number of correctly generated assignments (true positive, TP), the number of incorrectly generated assignments (false positive, FP), and the number of reference assignments that were not generated (false negative, FN). The performance of a generated alignment was assessed by its precision ($\text {TP}/\left( \text {TP} + \text {FP}\right)$), recall ($\text {TP}/\left( \text {TP} + \text {FN}\right)$), and F-score ($2*\ \text {precision}*\ \text {recall}\ /\ (\text {precision} + \text {recall})$). We also report the average performance measures over all source coding systems in each reference set (excluding reflexive alignments).

## Results

The VaccO ontology contained 321 vaccine classes with 706 terms (Table 4) including 206 classes for vaccine products, and 36 for common pharmacological groups and auxiliary classes corresponding to immunization targets (e.g., *Pertussis vaccines*), administration route (e.g., *Oral vaccines*), and vaccine strategy (e.g., *Attenuated vaccines*). Among the 497 classes for ingredients were 310 active ingredients, 170 excipients, and 21 adjuvants (some ingredients serving multiple roles). Classes for nine vaccine strategies with 34 terms were created: *Live/attenuated, Conjugated, Subunit, Inactivated, Polysaccharide, Recombinant, Synthetic, DNA*, and *Toxoid*. The 104 classes for pathogens contained 863 English terms. Pathogens were categorized by their biological domain in 56 classes for *Bacteria*, 42 for *Viruses*, and 6 for *Protozoa*, including 42 classes for pathogen strains. VaccO defines 49 classes for diseases with 759 terms, 30 valence classes with 71 terms, and 9 classes for administration routes with 23 terms.

**Table 4:** Number of classes and terms in the VaccO ontology

Fundamental class	Classes	Terms
Ingredient	497	505
Vaccine	321	706
Pathogen	104	863
Disease	49	759
Valence	30	71
Strategy	9	35
Route	9	23
Total	1,019	2,962

### Automatic code alignment

Table 5 shows the performance results of our alignment algorithm with different similarity methods in the two reference sets. These results were generated with a similarity threshold of 0.1, which had the highest average F-score over all alignments when we varied the threshold between 0 and 1 in steps of 0.1 (Table 6).

**Table 5:** F-scores of our alignment algorithms with a threshold of 0.1

Reference set: Vactype								
Method	Vactype	Venetia	Pedia	Pedia-it	SIDIAP	BIFAP	Average	CI
Tokens	1.000	0.652	1.000	0.364	0.305	0.372	0.539	0.355-0.805
Metamap	1.000	0.316	1.000	0.364	0.336	0.491	0.501	0.344-0.771
Classes	0.988	0.711	1.000	1.000	0.895	0.739	0.869	0.728-0.948
Equivalence	1.000	0.545	1.000	0.800	0.786	0.666	0.759	0.620-0.877
Properties	0.988	0.756	1.000	1.000	0.918	0.856	0.906	0.808-0.971
Reference set: ATC								
Method	ATC	MeSH	CHV	CVX	NDF-RT	NDF	Average	CI
Tokens	0.991	0.696	0.717	0.581	0.889	0.882	0.753	0.654-0.849
Metamap	0.932	0.533	0.808	0.966	0.780	0.788	0.775	0.634-0.893
Classes	0.960	0.435	0.667	0.848	0.667	0.595	0.642	0.513-0.739
Equivalence	0.950	0.755	0.842	1.000	0.905	0.914	0.883	0.804-0.945
Properties	0.947	0.930	0.920	1.000	0.974	0.970	0.959	0.928-0.983

**Table 6:** Performance measures of our alignment algorithms with varying thresholds (micro-average on both reference sets)

Method	Measure	0.0	0.1	0.2	0.3	0.4	0.5	0.6	0.7	0.8	0.9	1.0
Classes	F-score	0.643	0.756	0.755	0.744	0.719	0.715	0.734	0.722	0.728	0.717	0.713
	Precision	0.592	0.745	0.745	0.746	0.747	0.756	0.923	0.939	0.967	0.970	0.968
	Recall	0.816	0.771	0.770	0.751	0.716	0.709	0.644	0.629	0.624	0.604	0.599
Metamap	F-score	0.504	0.638	0.492	0.453	0.441	0.432	0.377	0.367	0.370	0.370	0.370
	Precision	0.487	0.793	0.798	0.800	0.818	0.814	0.861	0.926	0.943	0.943	0.943
	Recall	0.828	0.590	0.426	0.381	0.367	0.357	0.285	0.279	0.277	0.277	0.277
Properties	F-score	0.847	0.932	0.932	0.932	0.920	0.920	0.879	0.875	0.870	0.857	0.857
	Precision	0.812	0.944	0.944	0.946	0.947	0.947	0.949	0.958	0.962	0.968	0.968
	Recall	0.952	0.923	0.923	0.921	0.897	0.897	0.828	0.813	0.802	0.781	0.781
Tokens	F-score	0.557	0.646	0.631	0.634	0.370	0.346	0.249	0.160	0.160	0.160	0.160
	Precision	0.538	0.788	0.845	0.895	0.877	0.888	0.952	0.950	0.933	0.933	0.933
	Recall	0.834	0.618	0.591	0.576	0.269	0.244	0.156	0.094	0.094	0.094	0.094

The F-scores of the reflexive alignments were higher than 0.99 on the Vactype reference set and higher than 0.93 on the Atc reference set. The reason for the slightly lower performance on the Atc reference set is that codes for residual classes cannot be represented in OWL2 (e.g., J07BX with descriptor ‘Other viral vaccines’) and some ATC codes are defined without reference to specific vaccine properties (e.g., J07 for ‘VACCINES’, J07BC20 for ‘Combinations’). Overall, the reflexive mapping results indicated that the intermediate representations are capable of representing the descriptors of the target coding systems.

The baseline methods Tokens and Metamap performed poorly in the Vactype reference set with non-English descriptors because they were not designed to deal with multilingual input. On the Atc reference set, with only English code descriptors, their performance was higher. The other three methods, which used the multilingual VaccO dictionary, performed better on the Vactype reference set, with method Properties performing best for each source coding system (average F-score 0.91).

The performance was generally higher on the Atc reference set than on the Vactype reference set. Only method Classes performed better in the Vactype reference set, because a large variety of properties was used in the code descriptors for the same vaccine groups in the Atc reference set (e.g., ‘Flu vaccine’ vs. ‘Influenza virus vaccine’). These different descriptors were represented by different sets of VaccO classes, resulting in little similarity. The performance of methods Equivalence and Properties was less vulnerable to the variety of descriptions. Overall, method Properties performed best (average F-score 0.96) in the Atc reference set.

With a threshold of 0.1, the F-score of method Properties averaged over all alignments in both reference sets was 0.93, with a precision of 0.94 and a recall of 0.92. A threshold of 0.0 decreased precision to 0.81 and increased recall to 0.95 (F-score 0.85). A threshold of 1.0 increased precision to 0.97 and decreased recall to 0.78 (F-score 0.86).

### Error analysis

We analysed the errors made by method Properties (with a similarity threshold of 0.1) to identify remaining problems. For each pair of source and target coding systems, we considered all alignment errors. If there were more than 10 FP or FN errors we sampled 10 FP errors and 10 FN errors. The causes of a total of 64 errors were analysed and categorized.

The largest error source was the incorrect identification of classes in the code descriptors, mostly in the multilingual Vactype reference set (Table 7). These errors were caused by missing or ambiguous terms in the ontology dictionary. A second source of error in the Vactype reference set, was the lack of contextual knowledge in VaccO about the availability of vaccines. This knowledge had been used in creating the Vactype reference alignments, e.g., knowledge that only acellular vaccines are authorized was used to assign the source code of ‘Pertussis vaccine’ to the target code ‘Acellular pertussis vaccines’. The lack of contextual knowledge gave rise to the low performance of all methods in the Venetia source coding system. Thirdly, incomplete representation in the similarity method was a large error source in the Atc reference set. This includes errors where two target codes are semantically identical (e.g., ATC codes J07B for ‘Viral vaccines’ and J07BX for ‘Other viral vaccines’), where properties in the code descriptor do not correspond to classes in VaccO (J07AH06 for ‘meningococcus B, outer membrane vesicle vaccine’), or where codes are not defined based on specific vaccine properties (J07 for ‘VACCINES’, J07BC20 for ‘combinations’).

**Table 7:** Error analysis of automatic code alignment using the Properties method with a threshold of 0.1

	Vactype		Atc			
	FN	FP	FN	FP	Total	%
Incorrect class identification	14	6	4	1	25	39.0
Lack of contextual knowledge	7	14	0	0	21	32.8
Incomplete representation	1	0	12	5	18	28.1

### Web applications

Three web applications accompany the VaccO ontology. Application *Analyse* allows the user to enter a vaccine description and displays the identified classes, compiled DL-expression, and property values (similar to Fig. 3). Application *Selector* analyses a user-provided vaccine coding system, and enables the user to select codes based on their VaccO vaccine properties. Application *Alignment* allows the user to upload two arbitrary vaccine coding systems and generates and displays an alignment between them using the algorithm described above.

## Discussion

This article described VaccO, an application ontology for representing vaccine descriptions, and an algorithm for the automatic alignment of vaccine codes between general clinical and database-specific vaccine coding systems using multilingual code descriptors.

The alignment of vaccine coding systems presents three major difficulties: multilingual code descriptors, the use of different properties to describe the equivalent vaccine classes (e.g., by disease as in ‘Flu vaccine’ or by pathogen as in ‘Influenza virus vaccine’), and differing granularities of the source and target coding system. Our reference sets presented these difficulties by comprising code descriptors in English, Spanish, Italian, and Catalan, and contained general medical coding systems, drug coding systems, and custom database coding systems. The balance between precision and recall of the Properties method can be shifted by changing the similarity threshold. A lower threshold that increases recall can help when the automatically generated alignments are subsequently manually validated, as removing false-positive alignments generally is less effort than manually detecting missing false-negative alignments.

The Properties method allowed the creation of alignments between coding systems using different languages using its multilingual dictionary. The method is robust to differing conceptualizations and granularities in the vaccine coding systems through the use of ontology reasoning and the normalization of properties. The lack of contextual (e.g., country-specific) knowledge in VaccO, incompleteness in representing or differentiating certain codes in the ontology, and incompleteness of the dictionary were the main error sources in the approach.

The accuracy of the VaccO ontology aims to match the accuracy of the vaccine descriptions in coding systems to best serve the purpose of creating code alignments. Furthermore, the VaccO ontology is agnostic of any specific vaccine coding system and designed to represent the descriptors of any vaccine coding system. This is why the ontology does not define any vaccine codes at all, but only auxiliary classes, classes representing common vaccine abbreviations, and vaccine products. Vaccine products are included in VaccO to derive their properties when comparing code descriptors based of products with descriptors of pharmacological groups. VaccO focuses on European vaccines with its integration of the Art57 DB. Integration of other vaccine vocabularies could be used to change the geographical focus (e.g., RxNorm [55] for the United States or databases implementing ISO standard for the Identification of Medicinal Products (IDMP) [56]).

The presented VaccO ontology and the VO ontology [17] are both models of the domain of vaccines. The two ontologies, however, are designed from different points of view: VO models vaccine products and their immunological properties, whereas VaccO models properties used to describe vaccines in coding systems. Classes in VaccO and VO coincide where vaccine descriptions correspond to immunological properties of vaccine products, e.g., with respect to pathogens and ingredients. Differences between VaccO and VO result from the following deviations in vaccine descriptions from the immunological properties of vaccine products:

- Vaccine descriptions can be based on derived properties, which are not represented in VO (e.g., diseases and vaccine strategies derived from pathogens and ingredients, respectively).
- A vaccine immunizes against a pathogen, whereas vaccine descriptions may use pathogens and their corresponding vaccine-preventable diseases interchangeably. This ambiguity conflicts in VO with the definition of the property used for immunization targets, *vaccine-immunization-against-microbe*. The ambiguity is resolved in VaccO by incorporating diseases and relating them to their pathogens, permitting both pathogens and diseases in the range of property *immunizes-against*, and making diseases and their pathogens interchangeable using OWL2’s property chains.
- Vaccine descriptions imprecisely attach properties to vaccines, e.g., a vaccine can be described by a strategy, whereas the strategy is actually a property of one of its active ingredient. VaccO models equivalences between such imprecise descriptions again using property chains.

The therapeutic role of a drug is usually treated as an intent in biomedical ontologies. However, the differentiation between the intended and factual therapeutic role is unessential for representing descriptors of vaccine coding systems (e.g., all 2018 flu vaccines are categorized as *Influenza vaccine* even if not all instances immunize against the disease), and the property *immunizes-against* represents only the intended therapeutic role.

VaccO was designed as an application-ontology for our code alignment algorithm. The algorithm did not require the integration of VaccO with other ontologies such as VO or an upper-level ontology. But VaccO is based on the OWL2 standard, which facilitates a technically simple integration with other ontologies when required.

## Conclusion

The proposed method Properties for aligning vaccine coding systems performed excellently on a wide range of vaccine coding systems using different languages, which suggests broad applicability of the approach. The alignment method demonstrated the use of an application ontology to identify and represent vaccine descriptions, and the use of an ontology reasoner to comparing them. The automatic alignment of vaccine coding systems can accelerate the readiness of EHR databases in collaborative vaccine studies. The use of VaccO for the extraction of vaccine-related information from other free-text resources, e.g., scientific literature, spontaneous reports, or public news, requires further investigation.

## Data Availability

The VaccO ontology with three web applications and the source code under the CC-BY and GNU AGPL-3 licenses is available under https://app.vac4eu.org/vacco/.

## Footnotes

1 To improve readability of the DL expressions, we omit the existential operator (some) in the notation because no universal object property restrictions are used in VaccO. We use that as a synonym of and in the context of property restrictions. Class names are capitalized, names of object properties have lowercase names, and DL-keywords are underlined.
